# A Treatise on Intra-cranial Diseases; Inflammatory, Organic, and Symptomatic

**Published:** 1884-04

**Authors:** 


### BOOK NOTICES.

A Treatise on Intra-cranial Diseases; Inflammatory, Organic, and Symptomatic. Pp. 312. By Charles Porter Hart, M. D. Philadelphia: Hahnemann Publishing House, 1884.

Dr. Hart recently gave us a work on the diseases of the *nervous system.* This he now supplements by one on *Intra-cranial Diseases.* Both are good works, and useful additions to any one's library.

In the present work, we have, first (as was necessary to a proper understanding of the subsequent chapters on diseases), chapters on the *cerebral functions,* so far as known; next come brief, clear descriptions of the *cerebral diseases*—anæmia, hyperæmia, thrombosis, embolism, softening, sclerosis, tumors of, etc. These cerebral and nervous diseases are those whose pathology is especially scant and uncertain; they generally offer a field for the wildest empiricism.

We are glad therefore to observe how closely our author adheres to the rigid (and hence successful) homœopathic method of prescribing. Even in *insomnia,* where the temptation to use chloral, etc., is so pressing, we have given us the truth—to the exclusion of empirical nonsense.

